# Evaluation of self-ligation of the spermatic cord and vas deferens in open orchiectomy of healthy adult crossbreed dogs: clinical assessment, infrared thermography, and inflammatory biomarkers

**DOI:** 10.1590/acb412026

**Published:** 2026-04-17

**Authors:** Maurícia Elaine Pereira de Souza, Brunno Valença Oliveira, Ana Carolina Anchieta Adriano, Sarha Jane Evangelista Moura, Ed Johnny da Rosa Prado, Janice Keslen Neumann, Vinicius dos Santos Rosa, Fernando do Carmo Silva, Sandro de Vargas Schons, Tadeu Filipe de Oliveira da Costa, Juliana Targino Silva Almeida e Macedo, Brenda Miranda Dias Januário, Ivan Felismino Charas dos Santos

**Affiliations:** 1 Universidade Federal de Rondônia – Medicina Veterinária – Rolim de Moura (RO) – Brazil.; 2 Universidade Federal dos Vales do Jequitinhonha e Mucuri – Instituto de Ciências Agrárias – Medicina Veterinária – Unaí (MG) – Brazil.; 3 Universidade Estadual Paulista – Faculdade de Medicina Veterinária e Zootecnia – Botucatu (SP) – Brazil.; 4 Universidade Federal de Rondônia – Programa de Pós-Graduação em Biologia Experimental – Porto Velho (RO) – Brazil.

**Keywords:** Hemorrhage, Hemostasis, Ligation, Thermography

## Abstract

**Purpose:**

To evaluate the self-ligation of the spermatic cord and vas deferens in healthy adult dogs undergoing open orchiectomy, through clinical assessment, evaluation of acute inflammation, and surgeon satisfaction.

**Methods:**

Twenty adult male crossbreed dogs were included in the study and were randomly assigned to two groups of ten animals each. The SCT group underwent spermatic cord ligation using absorbable suture material, whereas, in the SCVT group, hemostasis was achieved through self-ligation involving the spermatic duct and vas deferens. The evaluated variables included clinical parameters, acute inflammatory biomarkers, inflammation at the surgical knot site via infrared thermography, surgical time, intraoperative bleeding, and surgeon satisfaction by using standardized scoring systems.

**Results:**

Surgical time was significantly shorter in the SCVT group compared to the SCT group (*p* = 0.021). The total leukocyte count was significantly higher (*p* = 0.01) in the SCVT group 24 hours after surgery.

**Conclusion:**

Both techniques proved to be safe and effective for open orchiectomy in healthy, medium-sized, crossbreed adult dogs. The SCVT was faster to perform, but it elicited a greater acute inflammatory response, as indicated by the increased total leukocyte count.

## Introduction

Orchiectomy is the most performed surgical procedure in small animals’ veterinary surgical practice as a method of contraception in male dogs and cats and consists of the surgical removal of the testicles^1-3^. The procedure is commonly indicated for hormone-mediated conditions such as prostatic diseases, adenomas, and perineal hernias, as well as for various undesirable behavioral changes such as territorial marking^4,5^. Other indications for orchiectomy include congenital abnormalities and endocrine disorders^5,6^.

The disadvantage of orchiectomy is primarily related to surgical complications that may arise from inadequate technique, with intraoperative and postoperative bleeding being the most common. One of the main approaches to prevent bleeding is the ligation of the spermatic cord using suture materials or vascular sealing devices^2,7,8^.

The ligature of the spermatic cord with suture material (SCT) is widely used in orchiectomies in dogs and cats and is broadly accepted as the standard procedure^9^. However, this technique has several disadvantages, including loosening of the ligatures or inadequate vessel occlusion, which may lead to intraoperative or postoperative bleeding; foreign body reactions; and the cost of suture materials^9,10^. Additional disadvantages include hematoma formation and postoperative pain, which can negatively affect the animal’s welfare and may require the implementation of enhanced pain management protocols^10,11^.

Given these disadvantages, it is necessary to investigate surgical techniques that minimize the risk of complications and postoperative discomfort in dogs undergoing orchiectomy. In this context, the use of autologous structures such as the spermatic cord and vas deferens for vascular ligation during open orchiectomy is highly valuable. However, studies evaluating this technique remain scarce in the veterinary literature^12-14^.

Therefore, the present study aimed to evaluate the self-ligation of the spermatic cord and vas deferens technique (SCVT) in healthy, medium-sized, crossbreed dogs undergoing open orchiectomy, through clinical evaluation; assessment of local and systemic inflammation using infrared thermography and inflammatory biomarkers, respectively; surgical time; intraoperative bleeding; and surgeon satisfaction. The study hypothesized that SCVT would induce a lower acute inflammatory response compared to SCT.

The surgical relevance of SCVT lies in its potential as a cost-effective alternative that utilizes autologous tissue structures, thereby eliminating the risk of suture material rejection.

## Methods

### Experimental environment and animal selection

The study was approved by the Animal Ethics Committee of the Universidade Federal de Rondônia (UNIR) (Approval No. 023-2022-A). All owners signed an informed consent form agreeing to the methodology and the inclusion of their animals in the study. The sample size was estimated using the “power.t.test” function from the “stats” package in R software, within the RStudio integrated development environment (Version 1.0.143, 2009–2016, RStudio, Inc.). Power and significance levels for a two-tailed hypothesis test were set at 0.80 and 0.05, respectively. Based on the largest sample size calculated under these parameters, the final sample consisted of ten animals per group, totaling 20 dogs.

The study was conducted in the mornings (7 a.m.) between May and January at UNIR, Rolim de Moura, Rondônia, Brazil (GPS coordinates: latitude 11°48’13”S, longitude 61°48’12”W, altitude 225 meters). All procedures were carried out in a controlled environment with ambient temperatures maintained between 20 and 22°C. The dogs were housed individually in cages throughout the study period.

### Inclusion and exclusion criteria

The dogs included in the study were up to date on vaccinations and deworming and were classified as ASA I according to the American Society of Anesthesiologists’ (ASA) scale, had no abnormalities on clinical examination or evidence of systemic disease, and had normal values for complete blood count, platelet count, alanine aminotransferase, total protein, albumin, creatinine, and urea.

Dogs with wounds or dermatitis on the scrotum or in the prescrotal regions; ectopic testicles; or were receiving medication and undergone any surgical procedure within 30 days before the study; or had a body condition score of 1–3 or 6–9 (on a 1 to 9 scale) were excluded from the study.

### Experimental procedure

Twenty healthy, adult, non-neutered, crossbreed, medium-sized male dogs were included in the study. The dogs were randomly assigned using Randomizer software into two groups of ten animals each, according to the hemostasis technique used during open orchiectomy:

- Group SCT: ligation of the spermatic cord using absorbable surgical suture;
- Group SCVT: self-ligation using the spermatic cord and vas deferens.

The order of surgeries within each group was also randomized using the Randomizer software.

### Anesthesia

All dogs were fasted for eight hours for food and one hour for water. Acepromazine (0.07 mg/kg) and pethidine (3 mg/kg) were administered intramuscularly 15 minutes before anesthetic induction. A 22G 1¼-inch (30 × 0.7 mm) catheter was inserted into the cephalic vein for the administration of maintenance fluids (10 mL/kg/h) using 0.9% saline solution.

Ketamine hydrochloride (10 mg/kg) and midazolam (0.5 mg/kg) were combined in a single syringe and administered intravenously. Anesthesia was maintained with half the initial dose of ketamine whenever respiratory or heart rate increased by more than 20%. Orotracheal intubation was performed after loss of jaw tone, and the tube was secured to the maxilla. A pulse oximeter was placed under the tongue to monitor heart rate and oxygen saturation, and electrocardiogram electrodes were attached to the thoracic and pelvic limbs.

### Surgical procedure

**Figure 1:**
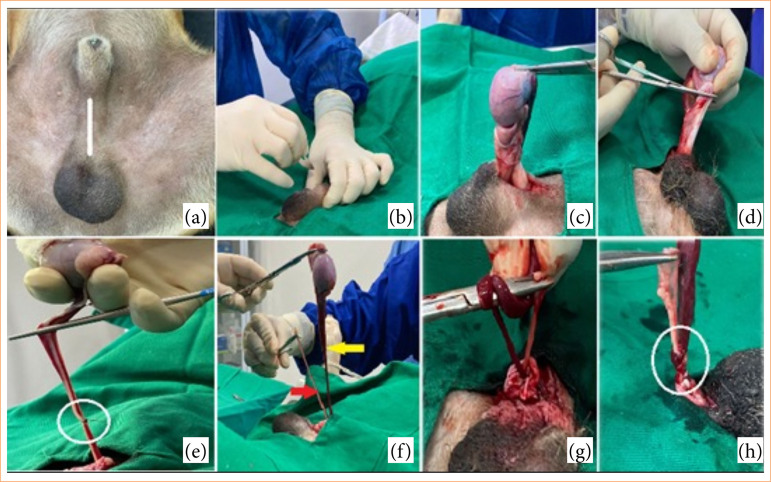
Photographic images illustrating (a) the wide hair clipped of the pre-scrotal and inguinal regions and the site of incision (white line); (b) the moment of skin incision over the testicle after its positioning in the pre-scrotal region and (c) subsequent exposure; (d) sectioning of the proper ligament of the testis and the cremaster muscle; (e) ligation of the spermatic cord (white circle) (SCT); (f) separation of the ductus deferens from the epididymis (red arrow) and pampiniform plexus and the testicular artery (yellow arrow) (SCVT); (g) ductus deferens tied over the vascular structures (pampiniform plexus and the testicular artery) using Mayo-Hegar needle holder; and (h) double surgeon’s knot finalized (white circle).

Source: Elaborated by the authors.

The dogs underwent hair clipping of the pre-scrotal and inguinal regions (Fig. 1a) using a 40-number trimmer, followed by pre-surgical antisepsis with 2% chlorhexidine and surgical antisepsis with 0.5% alcoholic chlorhexidine. The right testicle was positioned in the pre-scrotal region, and a standard pre-scrotal midline stab incision was made with a no. 22 scalpel, extending through the skin and common vaginal tunic to expose the testicle (Figs. 1b and 1c). The proper testicular ligament and cremaster muscle were dissected from the testicle using Metzenbaum scissors (Fig. 1d).

In the SCT group, the spermatic cord was clamped using Kelly forceps, followed by ligation with polyglactin 910 suture (2-0) (Fig. 1e). The spermatic cord was excised above the knot, and the testicle was removed.

In the SCVT group, the vas deferens was separated from the epididymis, and the pampiniform plexus and testicular artery were separated from the testicle using Hasted forceps (Fig. 1f). Hemostasis was achieved by tying the vas deferens with the vascular structures using a double surgeon’s knot with a Mayo-Hegar needle holder (Figs. 1g and 1h). These structures were excised approximately 2 cm distal to the knot.

In both techniques, the testicles were removed through the same incision. The subcutaneous tissue was closed using a simple continuous suture pattern with polyglactin 910 (2-0). The skin was sutured with a simple interrupted pattern using 2-0 nylon monofilament. Surgical time and any complications were recorded.

After surgery, dogs were relocated to individual kennels and monitored for signs of bleeding, hematoma, or swelling for up to 72 hours. For postoperative care, dipyrone (20 mg/kg orally) was administered every eight hours for five days. The surgical wound was cleaned with 0.9% saline solution every 24 hours until suture removal, which occurred 10 days post-surgery.

All anesthetic and surgical procedures were performed by the same individual with a minimum of two years of experience in these areas.

### Clinical evaluation

Clinical evaluation included the assessment of intraoperative and postoperative bleeding, hematoma formation, and local pain. Surgical time and surgeon satisfaction with the technique were also evaluated.

Intraoperative bleeding was scored according to Boezaart et al.^15^:

- 0: no bleeding;
- 1: mild bleeding, no compression required;
- 2: mild bleeding, compression required;
- 3: bleeding requiring occasional compression;
- 4: bleeding requiring frequent compression;
- 5: rapid and uncontrollable bleeding.

Surgeon’s satisfaction with the technique was measured using a five-point Likert scale:

- 1: very poor: certain postoperative knot dehiscence; technique deemed impractical for routine use;
- 2: poor: uncertainty regarding knot security; routine implementation undetermined;
- 3: neutral: no clear conclusion about knot security or practical applicability;
- 4: good: confidence in knot security, though some doubt regarding routine clinical application;
- 5: excellent: full confidence in knot security and applicability of the method in surgical practice.

Hemorrhage, hematoma, and pain on palpation of the surgical site were evaluated every 24 hours for three days and recorded as either present or absent. Surgical time was measured from the initial skin incision to the completion of skin suturing.

### Blood laboratory testing

A total of 6 mL of blood was collected by jugular venipuncture using 10-mL syringes and hypodermic needles (0.8 × 0.25 mm) for subsequent evaluation of leukocyte, platelet, and albumin levels. Three milliliters were transferred to siliconized tubes containing the anticoagulant ethylenediaminetetraacetic acid (EDTA) for leukocyte count, which was performed using the MAX CEL 200 hematology analyzer. Platelet count was conducted manually through the evaluation of Wright-stained blood smears examined under oil immersion (×100) using a light microscope.

The remaining 3 mL of blood was placed in siliconized tubes without anticoagulant but containing a clot activator and centrifuged at 5,000 rpm for 10 minutes to obtain serum. The serum was then transferred to Eppendorf tubes and stored at 4°C for later albumin measurement. Albumin concentration was determined using the semi-automated MAX Bio TOUCH analyzer by a colorimetric method, employing a veterinary-specific diagnostic kit (Labtest Diagnóstica S.A., Ref. 1007). All measurements followed the manufacturer’s instructions.

Leukocyte and platelet counts were assessed at the following time points: 1 hour before surgery (M_0_), and at 6 (M_6h_), 12 (M_12h_), 24 (M_24h_), and 48 hours (M_48h_) postoperatively. Albumin levels were measured at all time points except M0.

### Thermographic examination

The temperature at the knot site during open orchiectomy was measured using infrared thermography. For the thermographic assessments, the surgical room was maintained at the temperature of 22°C (± 1°C), with no exposure to sunlight, and only four persons were present, with restricted movement.

**Figure 2:**
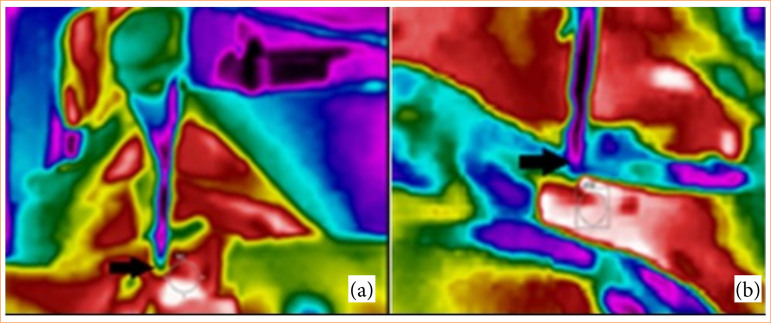
Infrared thermal imaging during the orchiectomy. Regions of knot (black arrows) in dogs of (a) spermatic cord with suture material and (b) spermatic cord and vas deferens technique.

Source: Elaborated by the authors.

Thermography was performed using a thermal imaging camera (FLIR Model E4; FLIR Systems), with images captured at 10 cm and perpendicular to the knot (Figs. 2a and 2b). The thermographic images were analyzed using FLIR Systems v.1.2 software. All examinations were conducted by the same person with experience in thermographic imaging. Temperature measurements were taken 10 minutes after the surgical knot.

### Statistical analysis

Data were processed using R software (version 4.3.1, 2023). Normality was assessed using the Kolmogorov–Smirnov’s test, and homogeneity of variances was verified with Levene’s test. The independent samples t-test was used to compare surgical time and thermographic temperatures. For continuous variables, those with a symmetric distribution were analyzed using repeated measures of the analysis of variance (ANOVA) (cross model), followed by multiple comparisons using the t-test. Surgeon satisfaction scores and intraoperative hemorrhage scores were compared between groups using the Mann–Whitney U test. A *p* ≤ 0.05 was considered statistically significant.

## Results

Dogs included in the study were crossbreed and had a body weight ranging from 10 to 24.5 kg (16.5 ± 4.7 kg) and ages ranged from 14 to 72 months (36 ± 18 months).

Only a single dose of anesthetic was administered intraoperatively in all animals. The SCVT was easy to perform, and no complications such as bleeding, pain, or hematoma were observed during orchiectomy or in the postoperative period in either the SCT or SCVT groups.

**Figure 3:**
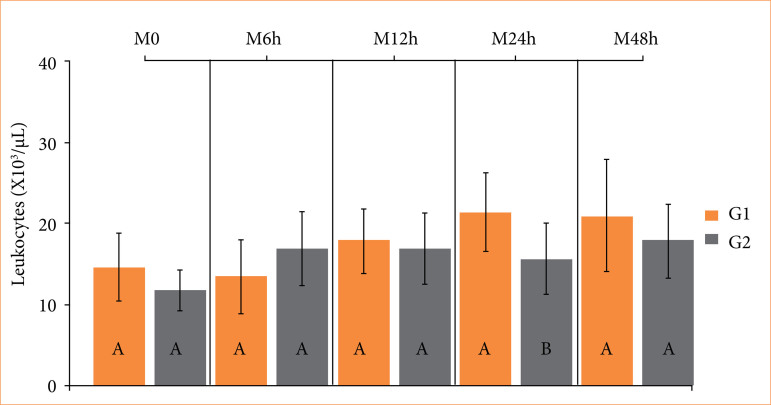
Boxplot of total leukocyte values in healthy adult dogs subjected to ligation of the spermatic cord using reabsorbed surgical suture (SCT) (G1), and self-ligation with spermatic cord and vas deferens (SCVT) (G2), at time-points: 1 hour before the surgical procedure (M_0_), 6 hours (M_6h_), 12 hours (M_12h_), 24 hours (M_24h_), and 48 hours (M_48h_) after the orchiectomy. Different uppercase letters indicate a significant difference (*p* < 0.05) between groups at each time point, according to the T-test. Reference values: 6–17 × 10^3^/µL.

Source: Elaborated by the authors.

**Figure 4:**
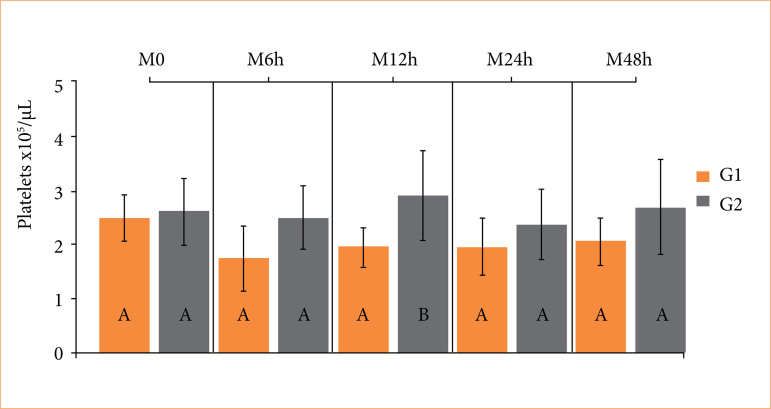
Boxplot of platelets values in healthy adult dogs subjected to ligation of the spermatic cord using reabsorbed surgical suture (SCT) (G1), and self-ligation with spermatic cord and vas deferens (SCVT) (G2), at time-points: 1 hour before the surgical procedure (M_0_), 6 hours (M_6h_), 12 hours (M_12h_), 24 hours (M_24h_), and 48 hours (M_48h_) after the orchiectomy. Different uppercase letters indicate a significant difference (*p* < 0.05) between groups at each time point, according to the T-test. Reference values: 2–10 × 10^5^/µL.

Source: Elaborated by the authors.

Surgeon satisfaction scores and bleeding scores did not differ between groups (Table 1). However, surgical time was shorter in the SCVT compared to the SCT (*p* = 0.021). Higher leukocyte counts (*p* = 0.01) were observed in the SCVT compared to the SCT 24 hours after surgery (Fig. 3). Platelet counts were higher (*p* = 0.03) in the SCT related to the SCVT 12 hours postoperatively, but these values remained within the reference limits (Fig. 4).

**Table 1:** Median with minimum and maximum values of satisfaction score; bleeding score, and mean ± standard deviation of surgical time in dogs submitted to ligation of the spermatic cord using reabsorbed surgical suture (SCT), and self-ligation with spermatic cord and vas deferens (SCVT) during open orchiectomy.

Variable	SCT group	SCVT group	*p* -value*
Satisfaction score (1–5)	5 (4–5)	5 (4–5)	0.14
Bleeding score (1–5)	1 (1–2)	1 (1–2)	0.163
Surgery time (minutes) without 10 minutes of infrared thermography	16.17 ± 2.12^A^	11.12 ± 2.19^B^	0.01

* *p*-values were determined by independent samples t-test for surgery time, and scores were compared with Mann–Whitney’s U test between groups.

Source: Elaborated by the authors.

**Figure 5:**
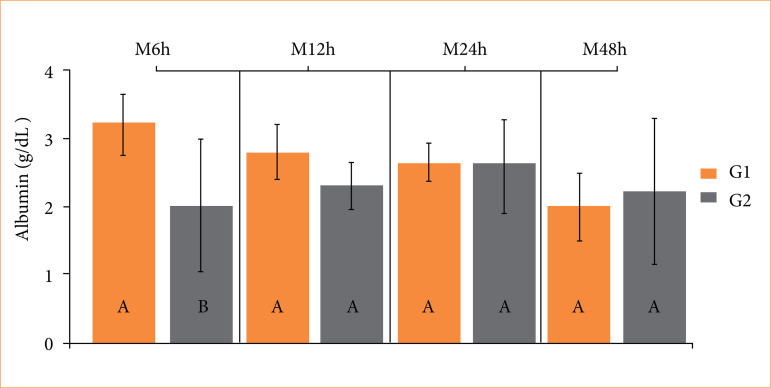
Boxplot of albumin values in healthy adult dogs subjected to ligation of the spermatic cord using reabsorbed surgical suture (SCT) (G1), and self-ligation with spermatic cord and vas deferens (SCVT) (G2), at time-points: 6 hours (M_6h_), 12 hours (M_12h_), 24 hours (M_24h_), and 48 hours (M_48h_) after the orchiectomy. Different uppercase letters indicate a significant difference (*p* < 0.05) between groups at each time point, according to the T-test. Reference values: 2.6–3.3 g/dL.

Source: Elaborated by the authors.

**Figure 6:**
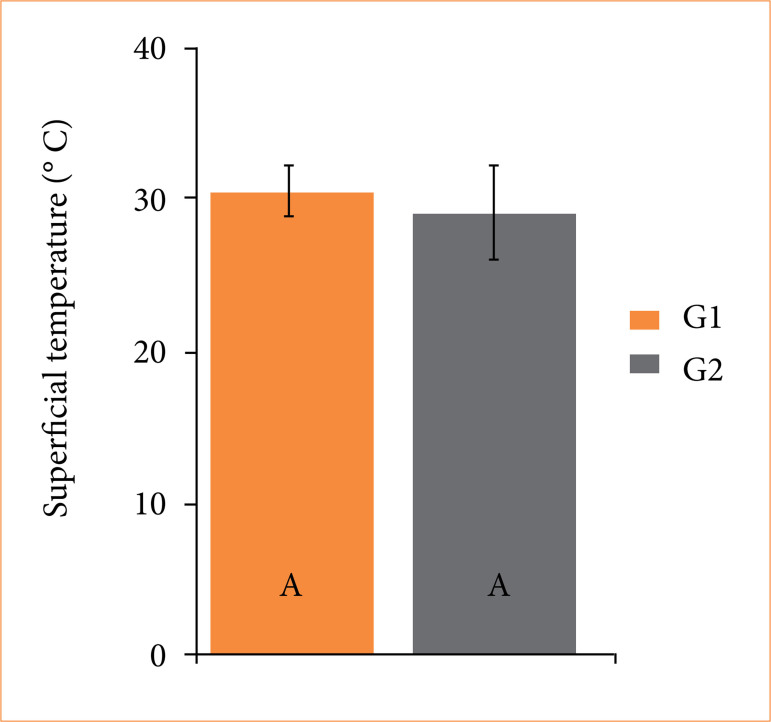
Boxplot of the surface temperature values (°C) of knot site of healthy adult dogs subjected to ligation of the spermatic cord using reabsorbed surgical suture (SCT) (G1), and self-ligation with spermatic cord and vas deferens (SCVT) during open orchiectomy. Different uppercase letters indicate a significant difference (*p* < 0.05) between groups at each time point, according to the T-test.

Source: Elaborated by the authors.

Albumin levels were lower (*p* = 0.01) in the SCT group compared to the SCVT group 6 hours after surgery, although values remained within the normal range (Fig. 5). No difference was observed in surface temperature at the knot site between both groups (Fig. 6).

## Discussion

The present study evaluated the hemostatic technique using self-ligation of the spermatic cord and vas deferens during open orchiectomy in healthy, medium-sized adult dogs, considering that the use of autologous structures for hemostasis is a viable alternative due to the absence of foreign body reaction. However, the study hypothesis was not confirmed, as the SCVT group showed higher total leukocyte counts compared to the SCT group 24 hours after the surgical procedure.

The SCVT technique used in this study is similar to that one described in cats and dogs. The practicality and rapid execution of the technique in both species were reported, supporting the findings of the present study^12-14,16^. According to Costa Neto et al.^12^, the technique is contraindicated in kittens due to the smaller diameter and fragility of the structures used for hemostasis. However, Miller et al.^11^ reported that SCVT can be applied in puppies and kittens, highlighting ongoing controversy regarding its use in young animals. Leal et al.^14^ reported the possibility of performing SCVT in small dogs weighing less than 5 kg and in cats; however, in dogs over 7 kg, the vascular ligature may loosen and cause intra or postoperative bleeding due to the increased thickness of the vas deferens and associated vessels14. In the present study, no complications, including bleeding, were observed, indicating that SCVT and SCT provided secure surgical knots.

Common postoperative complications associated with orchiectomy, including bleeding, infection, and abscess formation^1-3,11,13^, were not observed in the present study. However, Şenocak^13^ reported significant bleeding in dogs undergoing open orchiectomy with ligation of the vas deferens to the pampiniform plexus. Additionally, the study by Leal et al.^14^ highlighted mild complications, such as scrotal swelling three days after surgery in a dogs that underwent self-ligation using the vas deferens and vascular plexus.

In this study, acute inflammation was assessed through total leukocyte and platelet counts, albumin concentration, and surface temperature at the surgical knot site using infrared thermography. Surgical procedures induce tissue trauma and consequently trigger the release of inflammatory markers, which serve as indicators for validating surgical techniques^17-20^. On the other hand, albumin is considered a negative acute-phase protein and typically decreases during acute inflammation, unlike other inflammatory markers^21,22^. In the present study, total leukocyte counts were higher in dogs in the SCVT group 24 hours after surgery, indicating a greater acute inflammatory response in these animals. However, clinical evaluations after surgery revealed no signs of pain in dogs from either group. The SCT group exhibited lower albumin levels and higher platelet counts at 6 and 12 hours postoperatively, respectively. Nonetheless, the values remained within reference range.

Infrared thermography is a non-invasive method for assessing acute inflammation, as changes in the surface temperature of tissue regions indicate the presence or absence of an inflammatory process. This is associated with vasodilation, which occurs during inflammation and has been shown to be effective in detecting ocular and orthopedic inflammation in horses^23-26^. In the present study, no differences in temperature at the surgical knot site were observed between groups. However, Şenocak^13^ reported higher pre-scrotal temperatures in dogs undergoing spermatic cord ligation with suture material compared to those treated with self-ligation of the vas deferens to the pampiniform plexus. This fact was attributed to a greater inflammatory response caused by the suture material acting as a foreign body13.

Therefore, surgical techniques using autologous tissues may contribute to a reduced inflammatory response. Furthermore, the absence of suture material in the SCVT technique reduces surgical costs, which is especially important in contraceptive procedures performed in shelters managing large numbers of dogs, helping to avoid significant cumulative expenses^27^.

Both techniques were performed by the same surgeon. On average, the SCT technique took 5.05 ± 0.1 minutes longer than the SCVT technique. This difference was attributed to the greater ease of performing the SCVT method. The average time to perform the SCVT in the present study was 11 minutes and 12 seconds, while average surgical times reported in other studies ranged from 7 minutes and 48 seconds to 14 minutes and 55 seconds^13,14^. In contrast, the study conducted by Şenocak^13^ reported that the SCVT method took longer than the SCT technique, despite both being performed by the same surgeon. This was attributed to the surgeon’s initial lack of experience with the new technique.

Study conducted by Şenocak^13^ required four or five ligations between pampiniform plexus and vas deferens to secure the knots. In contrast, the present study showed that a double surgeon’s knot was sufficient to prevent bleeding. This difference establishes the SCVT as a safe choice, even in dogs over 7 kg with large spermatic cords.

This study demonstrated that the use of suture material remains a reliable hemostatic method for canine open orchiectomy. However, the SCVT proved to be equally effective, with surgeons reporting comparable satisfaction and bleeding scores. Moreover, the SCVT resulted in shorter surgical times compared to the SCT method and eliminated the need for suture material, which was an additional advantage that reduced the risk of knot dehiscence. It is emphasized that the choice between techniques will primarily depend on the veterinarian surgeon’s preference and experience.

The potential risk of arteriovenous fistula as a complication of self-ligation using the spermatic cord and vas deferens in canine orchiectomy was not assessed in this study. Further studies are needed to evaluate the long-term outcomes and compare them to fully determine the safety of the SCVT technique.

The limitation of this study was the absence of comparisons of pro-inflammatory interleukins (IL-1β, IL-6, tumor necrosis factor-α) due to budgetary constraints. These cytokines bind to target cell receptors and promote immune cell activation and recruitment to the site of inflammation. Another limitation was the lack of long-term follow-up to assess potential complications and fully validate the safety of the evaluated SCVT.

## Conclusion

The SCT and SCVT can be used as viable methods for hemostasis during open orchiectomy in healthy, medium-sized adult dogs. However, the SCVT technique is safe, cost-effective, and faster to perform, although it elicits a greater acute inflammatory response, as indicated by leukocyte counts. Furthermore, the SCVT technique utilizes only autologous material, thereby eliminating the risk of suture material slippage or rejection.

## Data Availability

All data sets were generated or analyzed in the current study.
